# I'm alone but not lonely. U-shaped pattern of self-perceived loneliness during the COVID-19 pandemic in the UK and Greece

**DOI:** 10.1016/j.puhip.2021.100219

**Published:** 2021-11-27

**Authors:** Alessandro Carollo, Andrea Bizzego, Giulio Gabrieli, Keri Ka-Yee Wong, Adrian Raine, Gianluca Esposito

**Affiliations:** a Department of Psychology and Cognitive Science, University of Trento, Italy; b School of Social Sciences, Nanyang Technological University, Singapore; c Department of Psychology and Human Development, University College London, London, UK; d Department of Criminology, Department of Psychiatry, Department of Psychology, University of Pennsylvania, USA; e Lee Kong Chian School of Medicine, Nanyang Technological University, Singapore

**Keywords:** Machine learning, COVID-19, Lockdown, Loneliness, Global study, Mental health

## Abstract

**Objectives:**

In the past months, many countries have adopted varying degrees of lockdown restrictions to control the spread of the COVID-19 virus. According to the existing literature, some consequences of lockdown restrictions on people's lives are beginning to emerge yet the evolution of such consequences in relation to the time spent in lockdown is understudied. To inform policies involving lockdown restrictions, this study adopted a data-driven Machine Learning approach to uncover the short-term time-related effects of lockdown on people's physical and mental health.

**Study design:**

An online questionnaire was launched on 17 April 2020, distributed through convenience sampling and was self-completed by 2,276 people from 66 different countries.

**Methods:**

Focusing on the UK sample (N = 325), 12 aggregated variables representing the participant's living environment, physical and mental health were used to train a RandomForest model to estimate the week of survey completion.

**Results:**

Using an index of importance, Self-Perceived Loneliness was identified as the most influential variable for estimating the time spent in lockdown. A significant U-shaped curve emerged for loneliness levels, with lower scores reported by participants who took part in the study during the 6th lockdown week (p = 0.009). The same pattern was replicated in the Greek sample (N = 137) for week 4 (p = 0.012) and 6 (p = 0.009) of lockdown.

**Conclusions:**

From the trained Machine Learning model and the subsequent statistical analysis, Self-Perceived Loneliness varied across time in lockdown in the UK and Greek populations, with lower symptoms reported during the 4th and 6th lockdown weeks. This supports the dissociation between social support and loneliness, and suggests that social support strategies could be effective even in times of social isolation.

## Highlights

• Of the 12 health-related variables, Self-Perceived Loneliness was the trait most affected by the time spent in lockdown.
• Replication across Greece and the UK uncovered a U-shaped curve for Self-Perceived Loneliness over time spent in lockdown.
• Results support the difference between social support and Self-Perceived Loneliness .
• Campaigns with improved social support could be effective in preventing loneliness, especially in precise time periods.

## 1 Introduction

The 2019 SARS-CoV-2 (COVID-19) outbreak was declared as a pandemic by the World Health Organisation (WHO) on 11 March 2020. The number of positive cases worldwide at the time was 179,111 and deaths, 7,426 [1].

Globally, the months that followed saw a surge in the number of deaths and infection rates, which put further strain on the sanitary and economical balance of several countries. Fast forward to September 13th, 2020, the total number of confirmed COVID-19 cases at 28,637,952 and 917,417 deaths have since been recorded [2].

Expert concern for the mental health consequences of the current pandemic stems from the evidence that was obtained during smaller epidemics, such as SARS (severe acute respiratory syndrome), MERS (Middle East respiratory syndrome-related coronavirus), H1N1, and Ebola. From these previous health emergencies, short- and long-term effects on the healthcare workers' mental health, such as post-traumatic stress disorder (PTSD) [3,4], depression [5,6], anxiety [5], stress and burnout [3] symptoms were common [7]. There is evidence that healthcare workers are distressed from the epidemics, during and after emergencies, and that these effects also extend to the general population in the form of severe anxiety, post-traumatic stress disorder, depression and increased rates of substance abuse [[8], [9], [10], [11], [12], [13]]. Although some promising results from vaccine trials are starting to emerge, the novel and highly infective virus continues to force governments around the world to limit people's movements and, in some cases, re-adapt lockdown restrictions once again, as in the case of the UK on September 22nd, 2020.

Closing schools and universities, shutting non-essential businesses, enforcing working from home policies and online teaching, struggling with financial difficulties and leaving the house only for necessities have fuelled genuine and perceived health threats that have rapidly become ubiquitous for large populations worldwide. While restrictions have helped flatten the infection curve, legitimate concerns about the physical and mental health consequences have been raised. As such, this pandemic, as an extreme global stressor, has provided an unprecedented opportunity for researchers to investigate how several aspects of our personal life, and specifically our physical and mental health, are affected by prolonged isolation and restrictions.

Social isolation is one known threat to mental and physical well-being [14,15] and an established risk factor for mortality [[16], [17], [18]]. Social isolation is associated with poor sleep quality [19] and with an increased risk of cognitive decline [20]. The fact that our perception of self is ingrained in the social comparison with others [21] suggests that social isolation may not be an ideal situation for the development of our identity either. Latest COVID-19 studies of the first weeks of lockdown have already documented psychological distress, such as depression, anxiety, post-traumatic stress, social mistrust and insomnia in Italy [[22], [23], [24], [25]] and China [26,27], the two countries most severely hit by COVID-19 at the beginning of the pandemic, as well as Austria [28] and Switzerland [29]. The adopted COVID-19 lockdown restrictions inevitably disrupted also the routine daily activities of the involved populations [30]. In fact, it has been documented that home confinement is associated with increased levels of physical inactivity and sedentary life [31,32]. Such a sedentary lifestyle negatively affects people's wellbeing and poses an accentuated risk towards chronic health conditions, such as cardiometabolic disease [31,33]. With some preliminary results on COVID-19 restrictions, this paper aims to address the gap in the literature regarding the effects of time in lockdown on people's health. Against the aforementioned backdrop of existing physical and psychological consequences from lockdown, this study focuses on the physical and psychological constructs that are more affected by prolonged lockdown periods. Starting from the hypothesis that time in lockdown has an impact on people's mental and physical wellbeing, a data-driven machine learning approach was adopted to identify the most time-sensitive health-related index during home confinement. Subsequently, the weekly variations in the identified variable's scores were examined under a statistical approach. By doing so, the current paper aims to provide a scientific contribution and help governments in the design of possible future lockdowns and social support.

## 2 Methods

### 2.1 Questionnaire

A 20-min online survey (available in 8 languages) was administered through the website www.globalcovidstudy.com between 17 April 2020 and 10 July 2020 to participants aged 18 years and above who had access to the survey link. The system used the IP address information to prevent participants from submitting more than one survey. The survey was distributed using various social media channels (email, LinkedIn, Whatsapp, Instagram, Facebook and Reddit).

The survey was designed by the Global COVID Study team (see https://osf.io/4nj3g/ [34] for more details), in order to explore participants’ moods and behaviours. The battery of questionnaires consisted of 359 questions assessing 13 main domains.

10 domains were investigated using standardized questionnaires: Physical Activity [35], Sleep Quality [[36], [37], [38]], Empathy [39], Anxiety [40], Depression [41], Self-Perceived Loneliness [42], Social Suspicions and Schizotypal Traits [43,44], Aggression [45], Demographic Information (including: Living Conditions [46], together with other more general features, such as participants’ gender, sexuality, age, accommodation, living space, marital status, education, ethnicity, occupation, income brackets, family history of health conditions), Parenting Style [47].

The remaining domains were investigated with custom questionnaires: Special Educational Needs, and two domains targeting COVID-related aspects: Worries and Beliefs about COVID protection rules (questionnaire with multiple-choice answers), and Report of how COVID and lockdown restrictions have influenced own behaviours and stress (open ended questions). The study was approved by the UCL Institute of Education Research Ethics Committee on 8 April 2020 (REC 1331). The study was conducted following the Declaration of Helsinki. All participants accepted the informed consent and all data were anonymized before the analysis.

For this study, we focused on 10 out of the 13 main domains of the survey (see Table 1). Parenting Style and Special Education Needs were excluded because variables in these domains did not apply to the general population and considering them could increase the data loss, because all non-parents or people with no Special Education Needs would have been excluded from the analysis. As a consequence, this would make the study results lose representativeness in regard to the general population. The Report of how COVID and lockdown influenced behaviours and stress was excluded as it reported free-text anecdotal answers. All the remaining domains were included in the study because they applied to the general population.

**Table 1:** Variables that are computed to quantify participants’ mental and physical health and living environment during lockdown.

Score	Description	Reference	Domain	Cronbach's Alpha (C.I. 95%)
Mild Activity Difference	Difference between days of mild physical activity post- and pre- COVID-19 lockdown.	*International Physical Activity Questionnaire – Short Form* (IPAQ-SF, 6-items) [35]	Physical Activity	Not applicable
Mild Activity Time Difference	Difference between minutes of mild physical activity post- and pre- COVID-19 lockdown.	*International Physical Activity Questionnaire – Short Form* (IPAQ-SF, 6-items) [35]	Physical Activity	Not applicable
Moderate Activity Difference	Difference between days of moderate physical activity post- and pre- COVID-19 lockdown.	*International Physical Activity Questionnaire – Short Form* (IPAQ-SF, 6-items) [35]	Physical Activity	Not applicable
Sleep Quality	Self-reported sleep quality and quantity, where higher scores reflect better sleep quality.	*Pittsburgh Sleep Quality Index* (2-items) [36], *Epworth Sleepiness Scale* [38], *Subjective and Objective Sleepiness Scale* [37]	Sleep Quality	0.71 (0.66–0.75)
Empathy	Self-reported affective, cognitive, and somatic empathy, where higher scores reflect higher empathy.	*Cognitive, Affective, Somatic Empathy Scale* (CASES, 30-items) [39]	Empathy	0.85 (0.83–0.87)
Anxiety	Higher scores reflect higher anxiety.	*General Anxiety Disorder-7* (GAD-7) [40]	Anxiety	0.88 (0.87–0.90)
Depression	Higher scores reflect higher depression.	*Patient Health Questionnaire-9* (PHQ-9, 9items) [41]	Depression	0.84 (0.82–0.86)
Self-Perceived Loneliness	Higher scores reflect higher self-perceived loneliness.	*Loneliness Questionnaire* (LQ, 20-items) [42]	Self-Perceived Loneliness	0.93 (0.92–0.94)
Living Condi-tions/Environment	Higher scores reflect more chaotic home environments.	*Chaos, Hubbub, and Order Scale and Health* *Risk Behaviors* (CHAOS, 6-items) [46]	Demographic Information	0.63 (0.58–0.69)
Beliefs	Perceived effectiveness of government guidelines on social distancing, schools closing, face masks and gloves as protection. Higher scores reflect stronger beliefs.	Summed 9-items on COVID-19 beliefs	Worries and Beliefs	0.81 (0.78–0.84)
Schizotypal Traits	Higher scores reflect more schizotypal traits.	Schizotypal Personality Questionnaire–Brief [43]	Social Suspicions and Schizotypal Traits	0.85 (0.83–0.87)
Reactive-Proactive Aggression	Higher score reflects more aggression.	Reactive-Proactive Aggression Questionnaire [45]	Aggression	0.85 (0.83–0.87)

Except for the Worries and Beliefs questionnaire, the selected domains were investigated by standardized questionnaires, widely used in the existing literature. The Physical Activity questionnaire was used to derive three variables quantifying differences between Pre- and Post-COVID: Mild Activity difference in days, Mild Activity difference in minutes per day, and Moderate Activity difference in days. The items in the Worries and Beliefs questionnaire were common COVID-19 prevention measures (e.g., “washing your hands frequently”) and the participants were asked to answer whether or not, in their opinion, the measures were useful in order to stop the spread of the virus. Specifically, participants could respond using a 5-points Likert scale (from 1- “I really don't believe this. This is fake news” to 5- “I strongly believe this”). The nine items were eventually summed in order to have an aggregated total score.

The remaining questionnaires were processed following standardized procedures to obtain one variable for each domain. To summarize, 12 variables were derived from the survey and used for the subsequent analysis.

### 2.2 Participants

Participants for the study were recruited through convenience sampling and, eventually, a total of 2,276 people (aged 18 and above) from 66 countries completed the survey during lockdown. Respondents who, in the first question, did not give consent to treat their data and to participate at the survey (N = 32), with incomplete (N = 712) or missing data (N = 165), or who could not complete the survey within two days from their enrollment (N = 76) were excluded. To train the RandomForest, we chose not to consider the participants who took more than one day to complete the survey, because the process required the model to find patterns of dependency between the features and the amount of time spent in lockdown. Considering the fact that, in our hypothesis, the time in lockdown played a role in determining the variability of the selected features, by considering only the participants who completed the survey within the same day, we aimed at reducing possible confounds. Furthermore, participants who completed the survey from a country which was not their residence country were excluded from the study (N = 132). Considering the variety of lockdown measures across the world, this criterion was adopted in order to reduce possible confounds given by the type of restrictions adopted by individual countries. Another possible confound came from the fact that not all the governments decided to adopt lockdown restrictions against the pandemic and, when they did, different countries entered the lockdown on different dates. For these reasons, among the countries that adopted these restrictions, the new variable “Weeks in lockdown” - the time of survey completion - was computed for each participant. This was done by calculating the difference in weeks between the day in which each participant completed the questionnaire and the date in which measures of lockdown were introduced for the specific country of interest. Thus, participants were grouped and compared regardless of the specific date in which their countries decided to adopt restrictions, but uniquely by the amount of time spent in lockdown. Furthermore, data from participants that reported pre-existing psychological, neurological or hormonal conditions or disorders were not considered for further analysis (N = 182).

Within this pool of data, the UK and Greece samples were selected for the analysis conducted in this study and data from participants living in other countries were discarded. Specifically, UK and Greece samples were selected because: a) they presented the highest sample size; b) they had a clear date in which lockdown started; and c) they both covered at least the weeks 3–7 after lockdown. Considering that the data collection for the current study started on 17 April 2020, no data were available for the 1st and 2 nd weeks of the UK and Greek lockdowns. UK participants that completed the survey after week 9 of lockdown were excluded from the study (N = 30).

### 2.3 Data analysis

In order to investigate the role of prolonged Time spent In Lockdown (TIL) on modulating the effects of lockdown restrictions on a cross-sectional fashion, the study consisted of two parts. In the first part, we aimed at identifying which of the investigated variables was most sensitive to the TIL. In the second part, we adopted statistical methods to assess and characterize how the identified variable was modulated by the TIL.

#### 2.3.1 Identification of the most influential variable

**Fig. 1:**
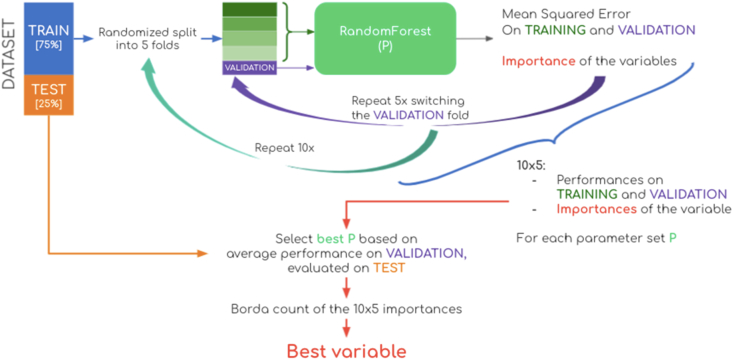
Design of the machine learning approach adopted in the current study. The UK dataset was divided into a training (75% of data; in blue) and a testing (25% of data; in orange) set. To train the model, the training set was randomly split into five folds. Four folds were given as input for the RandomForest's training on estimating the week of survey completion. The last fold (in violet) was used as a validation set to evaluate the training. Performances were evaluated by computing the Mean Squared Error (MSE) on the training and validation. Also, a ranking of feature importance was collected alongside. The same five folds were used five times to train and validate the model (violet arrow). The whole procedure, from the randomized split of the initial train partition, was repeated ten times, each time with five folds that were randomly selected (green arrow). From this standardized training procedure, 50 metrics of performance on training and validation in terms of MSE, together with 50 rankings of variables importance, were obtained for each parameter set (P) in the Random Forest. The optimal parameter P was eventually selected based on the average performance on validation, and the model was then evaluated on the testing set. A Borda count was computed on the rankings of variables importances to identify the best estimator on predicting the week of survey completion. (For interpretation of the references to colour in this figure legend, the reader is referred to the Web version of this article.)

Without any available literature to guide our hypothesis, to identifying the variable that is most influenced by the TIL we adopted a data-driven machine learning approach (see Fig. 1).

Machine learning was chosen because it allows ranking input variables used to create the model and because it allows processing high-dimensional data by design. Being a data-driven approach, it does not require any prior knowledge, and is therefore not limited by the scarce available literature on the topic, as a pure hypothesis-driven approach. As a consequence, machine learning is also capable of objectively highlighting research aspects which, if only based on prior knowledge, may be considered as less important. In particular, a RandomForest [48] regression model was trained to estimate the week in which each participant completed the survey, starting from the total scores of the 12 selected variables. The model creates an ensemble of decision trees based on the information in the input variables. It is critical to note that the estimation made by the model does not imply any causal relation between the input variables and target variable (the week in which the survey was completed). Conversely, the estimation relies uniquely on the analysis of the patterns of scores between the different variables and, for this reason, a cross-sectional design was still informative. The performance of the model was evaluated based on Mean Squared Error (MSE). The data used to train the RandomForest model were those of the 325 UK residents who were in the UK at the time of participation in this study. Initially, the dataset was partitioned into train (75% of participants) and test (25% of participants). The training process was repeated and evaluated several times on different randomized folds of the train dataset to optimize the number of decision trees and rank the variables based on their importance. A Borda count [49] was then computed on the rankings of variables obtained from each training iteration to identify the most important variable to estimate the week of survey completion. The optimal number of decision trees that emerged from the training was 200. The final model, with the optimal number of trees, was then trained on the whole train partition and evaluated on the test partition. The adopted training scheme is standardized and was derived from similar applications on bioinformatics [50] and public health [51].

#### 2.3.2 Statistical validation

In the second part of the study, we used a Kruskal-Wallis test to assess whether the most important variable (identified by the RandomForest model) significantly changes during the lockdown from weeks 3–8. In case of significant results, we adopted post-hoc Kruskal-Wallis tests to compare pairwise the 3rd week with the 4th to 8th weeks. The Bonferroni method was used to correct the significance level for multiple comparisons. In conducting statistical analyses, we first focused on the same set of participants used to train the RandomForest model, then we validated results on the dataset of participants from Greece.

## 3 Results

Eventually, the UK sample consisted of 325 participants (Gender: Female = 250 (76.92%), Male = 68 (20.92%), Non-binary = 3 (0.92%), Prefer not to say = 2 (0.62%), Self-identified = 2 (0.62%); Age (N = 1 participant did not report the age): Mean = 37.15; SD = 13.32; Ethnicity: Caucasians = 261 (80.31%), Asian = 34 (10.46%), Mixed background = 15 (4.61%), Hispanic Arab and Middle Eastern = 6 (1.85%), African = 2 (0.62%), Latin American = 3 (0.92%), Other ethnic group = 1 (0.31%), Prefer not to say = 3 (0.92%)), while the Greek sample counted 137 participants (Gender: Female = 102 (74.45%), Male = 35 (25.55%); Age: Mean = 36.25, SD = 10.92; Ethnicity (N = 2 participant, the 1.46% of the total, did not report the ethnicity): Caucasians = 128 (93.43%), Mixed background = 2 (1.46%), Other ethnic group = 2 (1.46%), Prefer not to say = 3 (2.19%)) (see Table 2).

**Table 2:** Number of participants from the United Kingdom and Greece by week. For each week, the demographic features, in terms of gender, average age, and accommodation, were reported.

Demographic Information	Week 3	Week 4	Week 5	Week 6	Week 7	Week 8	Week 9	Total
**United Kingdom**								
Sample size	36 (11.08%)	89 (27.39%)	69 (21.23%)	55 (16.92%)	60 (18.46%)	13 (4.00%)	3 (0.92%)	325
Gender: Female	30	69	51	40	46	11	3	250
Gender: Male	6	18	17	14	11	2	0	68
Gender: Non-binary	0	2	0	0	1	0	0	3
Gender: Prefer not to say	0	0	0	1	1	0	0	2
Gender: Self-identified	0	0	1	0	1	0	0	2
Average age	36.46	37.52	39.74	35.90	36.79	28.58	43.04	37.15
Accommodation: House (own)	12	40	28	23	18	3	3	130
Accommodation: House (rent)	1	6	9	5	4	2	0	27
Accommodation: Single bedroom flat (own)	1	2	1	1	2	0	0	7
Accommodation: Single bedroom flat (rent)	4	7	4	3	12	3	0	33
Accommodation: Double bedroom flat (own)	4	12	4	3	3	1	0	27
Accommodation: Double bedroom flat (rent)	2	8	8	5	7	0	0	30
Accommodation: Room in shared house (own)	0	1	2	2	0	0	0	5
Accommodation: Room in shared house (rent)	9	7	7	8	10	2	0	43
Accommodation: En-suit (own)	0	0	0	0	1	0	0	1
Accommodation: En-suit (rent)	1	1	2	1	1	0	0	6
Accommodation: Other	2	4	3	4	2	2	0	17
Accommodation: Not answered	0	1	1	0	10	0	0	12
**Greece**								
Sample size	15 (10.95%)	85 (62.04%)	29 (21.17%)	7 (5.11%)	1 (0.73%)	0 (0.00%)	0 (0.00%)	137
Gender: Female	13	58	26	4	1	0	0	102
Gender: Male	2	27	3	3	0	0	0	35
Average age	32.47	37.43	35.44	34.51	28.59	–	–	36.25
Accommodation: House (own)	3	28	7	5	0	0	0	43
Accommodation: House (rent)	1	4	3	0	0	0	0	8
Accommodation: Single bedroom flat (own)	1	4	2	0	0	0	0	7
Accommodation: Single bedroom flat (rent)	3	12	3	0	0	0	0	18
Accommodation: Double bedroom flat (own)	3	15	5	2	1	0	0	26
Accommodation: Double bedroom flat (rent)	2	10	6	0	0	0	0	18
Accommodation: Room in shared house (own)	0	1	0	0	0	0	0	1
Accommodation: Room in shared house (rent)	0	0	0	0	0	0	0	0
Accommodation: En-suit (own)	0	1	1	0	0	0	0	2
Accommodation: En-suit (rent)	0	1	0	0	0	0	0	1
Accommodation: Other	2	7	1	0	0	0	0	10
Accommodation: Not answered	0	2	1	0	0	0	0	3

Psychometric information about the study variables in the UK and Greece samples are detailed in Table 3.

**Table 3:** Distribution of scores across the variables adopted to train the RandomForest model. The table, divided by sample (UK and Greece) includes scores representing: Minimum (Min), First Quartile (Q1), Median, Mean, Third Quartile (Q3), Maximum (Max), and Standard Deviation (SD). Higher scores for Mild Activity Difference (in days), Mild Activity Time Difference (in min), and Moderate Activity Difference (in days) indicate an increased physical activity - either mild or moderate, in days or minutes - during the lockdown period compared to the pre-lockdown period. For Sleep Quality, Empathy, Anxiety, Depression, Self-Perceived Loneliness, Schizotypal Traits, and Reactive-Proactive Aggres-22 sion, higher scores stay for higher reported symptoms. For Living Condition/Environment, higher scores reflect a more chaotic living environment. For Beliefs, higher scores suggest higher confidence on the adopted measures against the spread of COVID-19.

Score	Min	Q1	Median	Mean	Q3	Max	SD
**United Kingdom**							
Mild Activity Difference (in days)	−7.00	−3.00	−2.00	−1.61	0.00	7.00	2.66
Mild Activity Time Difference (in min)	−780.00	−30.00	0.00	−15.17	10.00	210.00	66.88
Moderate Activity Difference (in days)	−7.00	−1.00	0.00	−0.19	1.00	7.00	2.44
Sleep Quality	5.00	12.00	15.00	14.70	18.00	23.00	3.75
Empathy	20.00	42.00	48.00	46.78	53.00	60.00	7.96
Anxiety	0.00	2.00	4.00	5.11	7.00	21.00	4.60
Depression	0.00	3.00	6.00	6.65	9.00	25.00	5.16
Self-Perceived Loneliness	20.00	32.00	39.00	40.64	47.00	70.00	10.78
Living Conditions/Environment	6.00	9.00	11.00	11.76	14.00	30.00	4.03
Belifs	16.00	34.00	38.00	37.22	40.00	45.00	4.26
Schizotypal Traits	0.00	2.00	4.00	5.35	8.00	20.00	5.35
Reactive-Proactive Aggression	0.00	3.00	5.00	5.74	8.00	23.00	4.23
**Greece**							
Mild Activity Difference (in days)	−7.00	−3.00	0.00	−0.72	1.00	6.00	2.57
Mild Activity Time Difference (in min)	−480.00	−15.00	0.00	3.43	20.00	510.00	77.83
Moderate Activity Difference (in days)	−6.00	0.00	0.00	−0.04	0.00	7.00	2.07
Sleep Quality	7.00	14.00	16.00	15.97	18.00	23.00	3.30
Empathy	29.00	41.00	46.00	44.85	50.00	60.00	6.45
Anxiety	0.00	1.00	3.00	4.28	6.00	20.00	4.36
Depression	0.00	2.00	4.00	5.27	7.00	22.00	4.15
Self-Perceived Loneliness	23.00	31.00	38.00	40.03	47.00	71.00	11.07
Living Conditions/Environment	6.00	10.00	11.00	12.07	14.00	24.00	3.55
Belifs	19.00	31.00	34.00	33.53	36.00	45.00	4.95
Schizotypal Traits	0.00	2.00	5.00	5.55	8.00	19.00	4.43
Reactive-Proactive Aggression	0.00	5.00	9.00	8.86	12.00	21.00	4.64

**Fig. 2:**
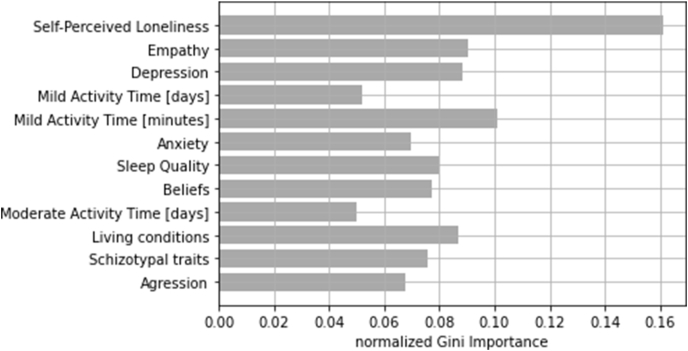
Average importance of the 12 health-related variables selected to train the RandomForest model on estimating the week of survey completion. Gini normalized importance values - an indicator of feature relevance - are obtained by computing a Borda count on the variables importance rankings on each iteration of a 10x5 cross-validations training scheme.

MSE on the training and the test partitions of the UK sample was 1.17 and 1.84 respectively and the feature with the highest importance was the Self-Perceived Loneliness (see Fig. 2).

**Fig. 3:**
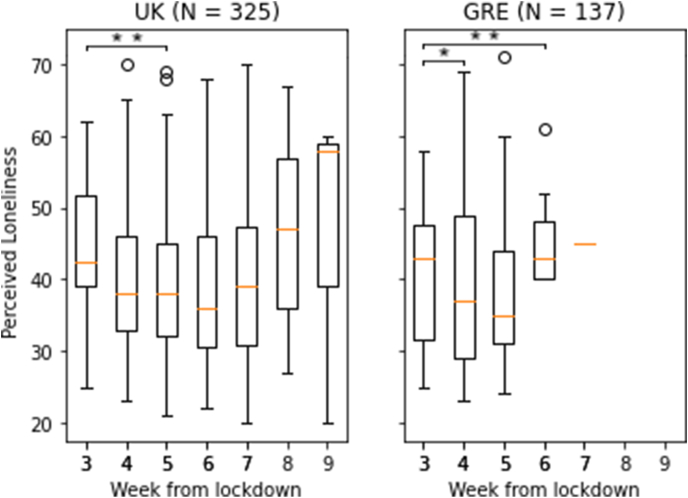
Cross-sectional U-shaped distribution of Self-Perceived Loneliness scores for each week for participants from the UK (N = 325; left) and Greece (N = 137; right). The orange line within each bar represents the median score for each week. Median was chosen over the mean as it is less influenced by extreme values - namely, outliers (represented in the picture by the circles). Week 7 for Greece has only the orange line, with no box, because only one participant took part in the study in that period. Weeks 8 and 9 for Greece do not have bars because no participant took part in the study during that period of time. (*p < 0.017; **p < 0.01). (For interpretation of the references to colour in this figure legend, the reader is referred to the Web version of this article.)

Notably, scores of Self-Perceived Loneliness decreased when comparing the reports from UK participants who took part in the study during weeks 3 to the ones that participated in week 6 of lockdown and subsequently increased when examining the reports from participants who were enrolled in the following weeks, returning to the initial values (see Fig. 3).

The Kruskal-Wallis test on the data of UK participants from the 3rd to 8th week confirmed that at least one week was statistically different from the others (H = 11.74, p = 0.039). We then compared the 4th, 5th, 6th, 7th and 8th week with the 3rd. Significant differences were found for the 6th (H = 6.91, p = 0.009) week, but not for the 4th (H = 6.30, p = 0.012), the 5th (H = 5.56, p = 0.018), the 7th (H = 3.88, p = 0.049) and the 8th (H = 0.33, p = 0.563) week.

The same procedure was repeated on participants from Greece, focusing only on the 3rd to 6th weeks, as only one participant completed the survey during the 7th week and none during the 8th week. The results confirmed that, between participants, Self-Perceived Loneliness significantly changes over weeks 3–6 (H = 8.89, p = 0.031), with significant differences between the 3rd and the 4th weeks (H = 6.30, p = 0.012) and between the 3rd and the 6th weeks (H = 6.91, p = 0.009). The difference between the 3rd and the 5th weeks (H = 5.56, p = 0.018) failed to survive the Bonferroni correction.

## 4 Discussion

The aim of this study was to assess the impact of the time spent in lockdown restrictions on people's mental and physical health. Although we adopted a rigorous methodology to train the predictive model, the achieved performances on train and test partitions are low: this outcome reflects the complexity of the psycho-social mechanisms that were at play during the lockdown period. In fact, different psycho-social dynamics could emerge in relation to the person's living environment, such as the city's dimension or the geographic area where they live. Aspects, these, that were not assessed in the initial questionnaire and that contribute to limit the generalizability of the current results. Another aspect that may limit the generalizability of the results is that the distribution of the sample’ demographic characteristics do not always follow the general population's ones. For instance, although the sample collected from Greece reflects the ethnic distribution of the general population, it does not reflect the general gender ratio [52]. The same can be said for the UK, with also some observed difference between the collected sample's ethnicity and the general population's one [53]. While the questionnaires aimed at quantifying a broad range of the aspects of interest, other aspects may not have been observed. Furthermore, the collected data were based only on self-report measures, and this may have led to inaccuracies (over- and under-reporting) when reporting symptoms or physical activity levels. Additionally, this study investigated the temporal variations of these mechanisms, whose effects on the observed variables might be even more difficult to identify. This is true, especially if considering the cross-sectional design of the study, which does not allow to infer the presence of a causality relation between variables. That said, one advantage of the machine learning approach is that it permits the identification of variables that are more sensitive to the time spent in lockdown, rather than a focus on predictive capability. The low performance does not affect the reliability of the ranking of the variables, which identified Self-Perceived Loneliness as the most sensitive variable.

This does not conflict with the literature on the impact of COVID-19 pandemic on people's health, which documented increased rates of psychological distress - mainly in regards to depression and anxiety symptoms [[54], [55], [56], [57]] - and decreased physical activity levels that pose long-term risk towards cardiovascular diseases [[58], [59], [60]]. To identify the most sensitive variable, the machine learning model relies on the variability of scores of such variable in relation to the time in lockdown. For this reason, Physical Activity variables (with Mild Activity Time Difference that was the second most time-sensitive variable), Depression (although it was the fourth most important variable for the model's performance) and Anxiety scores may have not been the most time-sensitive ones because of more stable (either high or low) levels of reported scores. Furthermore, it is likely that Self-Perceived Loneliness's scores vary to a larger extent in the short-term, during the first weeks of lockdown restrictions, than depression and anxiety's scores, which may change to a broader degree in the long-term [61]. Hence, our results are far from saying that the amount of physical activity, depression and anxiety are less affected by lockdown restrictions. Rather, they suggest that, during lockdown, physical activity, depression and anxiety scores do not fluctuate in relation to time in lockdown as much as Self-Perceived Loneliness.

In the study, reports of Self-Perceived Loneliness in the UK were particularly low for the participants who took part in the study during the 6th week after the start of the lockdown, before returning to initial values for the participants who compiled the survey afterwards. The pattern was replicated in the Greek sample, albeit in a smaller group of participants. This supports the fact that Self-Perceived Loneliness does capture a sensitive decrease during weeks 4–6 since the start of lockdown, independently, to a certain extent, from the specific rules that were adopted by the countries. Nevertheless, it is worth noting that the restrictions in the two countries were generally similar and the results may have been replicated for this reason. In fact, in both the UK and Greece, all non-essential movements throughout the country were banned. People were ordered to stay at home and avoid contact with each other. Schools, non-essential travels and businesses were shut.

The results of the study suggest that, in a period in which a large part of the global population was not allowed to see their close friends, partner, and family (of course, under the assumption that all people followed the rules), participants who took part in the study during weeks 4–6 reported lower levels of Self-Perceived Loneliness if compared to participants who completed the survey in the previous or subsequent weeks. This contrast between the objective isolation (in this case the one that was forced by lockdown restrictions) and the self-perception of loneliness (which in this case was perceived as lower) has been largely discussed in the existing literature [62,63]. It is believed that perceived social isolation represents a quantitative or (more often) qualitative mismatch between an individual's need for social support and the subjective evaluation of the social support that is obtained [64]. In other words, the feeling of loneliness, resulting from the perception of social isolation, seems to depend, more than on an objective condition of isolation, on a cognitive evaluation and perception of the social environment [65]. The reason behind the results of this study is not clear, but some hypotheses can be advanced. For instance, considering the definition of loneliness as a mismatch between desired and obtained social support, its low levels in the first weeks of lockdown could signify that people in that period of time were receiving the desired social support or even more of it in terms of quantity or quality. In the emerging literature about COVID-19, the number of friends and one's social support seem to play a protective role against the effect of lockdown on loneliness [66,67]. This said, a reduction in the levels of loneliness in the initial weeks of lockdown for both the UK and Greece may depend on other aspects whose impact was not assess in the current study. For instance, the number of reported cases may have played a role. In fact, after about one month of restrictions, the reported cases per day were diminishing [68] and this may have provided in the populations a sense of safety and collaboration that reduced the self-perception of loneliness. Another possible explanation is that, at least initially, restrictions were followed by people's appetite to meet online rather than in-person. The hunger for virtual activities which were facilitated by digital platforms, at least for the first weeks of lockdown, may have helped reducing the sense of loneliness in people's life.

Even though no certain explanation can be given for understanding the observed patterns of Self-Perceived Loneliness, the findings of this study support the idea that social isolation (as the objectively low social support) and loneliness (as the subjectively low social support) are different concepts, not necessarily linked to each other, as philosophers in the past centuries have largely pointed out. Having observed that lockdown restrictions have shortterm effects on people's feeling of loneliness and not knowing the real meaning behind these observed patterns, in our opinion, the design of possible future lockdown measures should be accompanied by the consideration of the role played by real and perceived social support for people's physical and mental well-being. In fact, the feeling of loneliness seems to be connected to the concept of Self [69], the person's cognitive functioning [63] and, in general, the mental and physical well-being of the individual [[15], [16], [17],^70^]. For instance, lonely people are more likely to suffer from depression [71,72], Alzheimer's disease [72,73], alcoholism [72,74,75], suicide [72,76], personality disorders [72] and sleep problems [72]. This set of evidence highlights the importance of taking into account the people's sense of loneliness when designing future lockdown restrictions or similar situations where social interaction is limited.

## 5 Conclusions

Existing studies have reported fluctuations in people's mental and physical wellbeing over the course of the pandemic and prevalence rates of loneliness, but not directly linked to the lockdown restriction period and duration. As countries around the globe continue to engage in new lockdowns now and in the future, a key aim of the study is the importance of assessing the short-term physical and mental health challenges and concerns that lockdown restrictions can bring on individuals and societies. This paper goes beyond prevalence rates but pinpointed how a range of variables across lockdown periods - by comparing data from two countries -, and within lockdowns by focusing the analysis on the effects of time spent in lockdown. The study built on past evidence to investigate a wider range of potential physical and mental health variables that could be at play in order to obtain a more holistic picture of what it means to experience lockdown restrictions. To do so, a data-driven approach was adopted to minimize any bias due to the lack of available literature on the topic. The results highlighted differences in levels of loneliness during lockdown periods by weeks in participants from the UK and Greece. In fact, loneliness was the most time-sensitive variable, even more so than physical activity, depression and anxiety. These results do not exclude a role of lockdown restrictions affecting symptoms of depression and anxiety or levels of physical activity, just that these symptoms do not vary across the same time period. Rather, Self-Perceived Loneliness emerged as a potentially important time-sensitive construct that deserves attention from policymakers now to help people better cope with lockdowns, or to at least know to expect fluctuations in feelings of loneliness during tight lockdown restrictions.

## Author contribution

Conceptualization: A.B., G.G., K.K.W., G.E.; Data curation: A.C., A.B., G.G., K.K.W.; Data analysis, Data interpretation, Writing: A.C., A.B.; Revision: A.C., A.B., G.G., K.K.W., A.R., G.E.; Supervision: G.E. All authors read and agreed to the published version of the manuscript.

## Ethics

This study was pre-registered (https://osf.io/4nj3g/) on 17 April 2021 and ethical approval for the COVID-19 Social Study was granted by the University College London Institute of Education Ethics and Review Committee in April 2020 (REC 1331; [34]). The study is GDPR compliant.

## Funding

This research is supported by 10.13039/501100001475Nanyang Technological University (Singapore) under the NAP-SUG grant to GE.

## Declaration of competing interest

The authors declare no conflict of interest.
